# Among key social determinants, connection associated with lowest odds of non-suicidal self-injury and suicidal ideation among Australian youth

**DOI:** 10.1177/00048674261438055

**Published:** 2026-04-09

**Authors:** Alysia M Robertson, Veronica Sheanoda, Paul Campbell, Emma Knight, Tegan Cruwys, Jason DE Proulx, Joanne A Rathbone, Katherine J Reynolds

**Affiliations:** 1 School of Medicine and Psychology, The Australian National University, Canberra, ACT, Australia; 2 Faculty of Education, The University of Melbourne, Melbourne, VIC, Australia

**Keywords:** Adolescence, young people, suicide, self-harm, social connectedness, socioeconomic factors, group belonging

## Abstract

**Objective:**

To test the relative importance of key social determinants (as specified in the Australian *National Suicide Prevention Strategy 2025–2035*) associated with suicidal ideation and non-suicidal self-injury in Australian youth.

**Methods:**

Representative data from the cross-sectional National Study of Mental Health and Wellbeing 2020–2022 were analysed for Australian youth aged 16–24 years. We used population-weighted multivariable logistic regression analyses to predict 12-month suicidal ideation and non-suicidal self-injury from several social determinants: household income, receipt of government pension/allowance payments, study/work engagement, experience of homelessness, household financial stress, urban residence and social connectedness.

**Results:**

Social connectedness was a statistically significant predictor in both models, such that a one-unit increase reduced the odds of 12-month suicidal ideation by 52% and non-suicidal self-injury by 55%. Other significant predictors were having ever experienced homelessness, which increased the odds of suicidal ideation by 116%, while each household financial stressor (e.g. not being able to pay bills on time) increased the odds of non-suicidal self-injury by 34%. No other social determinant was statistically significant in the multivariable models.

**Conclusion:**

Social connectedness was the only social determinant associated with lower odds of both suicidal ideation and non-suicidal self-injury in youth. Although further longitudinal studies are needed to confirm these benefits, our cross-sectional findings provide initial support for the National Suicide Prevention Strategy’s emphasis on strengthening social inclusion and economic security as key prevention strategies. Our findings highlight the importance of implementing and evaluating connectedness interventions for youth as a priority next step in suicide and NSSI prevention efforts.

## Introduction

Suicide is the leading cause of death for Australian youth (people aged 15–24 years) and, therefore, represents a critical public health priority (Australian Institute of Health and Welfare [AIHW], 2025a). Suicidal ideation (i.e. having suicidal thoughts) and non-suicidal self-injury (NSSI) – deliberate injury of oneself without the intention of dying (Nock, 2010) – are key markers of suicidal distress, a deep psychological pain that is even more widespread than and often a critical antecedent of suicide (AIHW, 2025d; Arya et al., 2024; Hamza et al., 2012; Klonsky et al., 2021). In this study, we examine these two markers of suicidal distress as distinct, but related, targets for the prevention of youth suicide.

Youth may be especially likely to engage in suicidal and NSSI behaviours, as adolescence and early adulthood often involve significant stressful life transitions (e.g. moving from school to university or work, moving out of home), as well as coinciding with the typical onset of most mental health conditions (Solmi et al., 2022). Initiatives that reduce suicidal distress in youth are, therefore, crucial as they can deliver ‘a triple dividend, with benefits for young people today, for the adults they will become, and for the next generation of children whom they will parent’ (Baird et al., 2025: 1945).

In recognition of the urgent need to address the burden and impact of suicide and NSSI in Australia, the Australian Government released the *National Suicide Prevention Strategy 2025–2035* (NSPS; National Suicide Prevention Office, 2025). The NSPS outlines a whole-of-government approach to coordinated suicide prevention efforts and prioritises social determinants as upstream prevention targets, in line with the prevention strategies of other countries (U.S. Department of Health and Human Services, 2024). It is structured around two key domains: (1) prevention of suicidal distress and (2) support for people experiencing suicidal distress and those who care for them.

Central to the NSPS is the acknowledgement that suicidal distress often arises as a result of *social determinants* interacting with a person’s individual risk factors (e.g. age, gender, exposure to trauma, mental health conditions and genetics). Social determinants can be defined as the varying structural, social and other non-medical factors that affect people’s daily lives and produce inequities in health within and between social groups (Kirkbride et al., 2024; Pirkis et al., 2024). The model provided in the NSPS closely aligns with the model outlined by Pirkis et al. (2024). The NSPS social determinants include income and social protection, employment and job security, working life conditions, housing and basic amenities, structural equality, education, early childhood development, environment and access to quality health care.

One factor that has received considerable attention in previous work is social connectedness. Research indicates that social connections are protective against suicidal thoughts and behaviours, while loneliness and social isolation are robust risk factors (Blázquez-Fernández et al., 2023; McClelland et al., 2020; Motillon-Toudic et al., 2022; Shoib et al., 2023). This relationship has been demonstrated using diverse study designs, such as longitudinal studies (Arango et al., 2024; Gunn et al., 2018), randomised trials (Skopp et al., 2023; Van Orden et al., 2021) and case–control studies (Wu et al., 2013). Key theoretical frameworks also highlight the role of social isolation as a risk factor for suicide and NSSI. Of note, the Interpersonal Theory of Suicide (Van Orden et al., 2010) positions thwarted belongingness and perceived burdensomeness as key predictors of suicide.

We focus on examining the relative importance of the social determinants of suicide and NSSI listed in the NSPS for two key reasons. First, social determinants may be more modifiable and effective targets for intervention than individual risk factors of suicide and NSSI. For instance, it is possible to improve a person’s social connections or access to basic amenities, but impossible to change their genetics or history of exposure to trauma. Second, the individual risk factors in the NSPS model have already been supported by high-quality empirical evidence (e.g. see reviews by Evans et al., 2004; Hawton et al., 2012; Richardson et al., 2024), whereas research on social determinants has received comparatively less research attention (Franklin et al., 2017). Interest in the social determinants of suicide is growing (Gallagher et al., 2025; Na et al., 2025); however, the existing evidence base would benefit from greater nuance to effectively inform youth prevention strategies. Specifically, three key gaps limit our understanding of how social determinants relate to youth suicide and NSSI risk.

First, while *individual* social determinants have been examined in relation to suicide and NSSI (e.g. the body of work on social connectedness outlined above), few studies have examined multiple social determinants in the same statistical model to determine their relative importance. One notable exception to this is the Youth Self-Harm Atlas study (Hielscher et al., 2022, 2024), which investigated the variability of self-harm (both suicidal and non-suicidal) across Australia for adolescents aged 12–17 years, as well as examining key risk and protective factors (both individual and social determinants).

Second, few studies have directly compared social determinants of suicidal ideation (and other suicidal experiences) and NSSI within the same sample, despite evidence that these are distinct phenomena with different risk profiles (De Neve-Enthoven et al., 2024; Mars et al., 2014; Nock, 2010; Wichstrøm, 2009; Wong et al., 2007). Understanding whether social determinants associate similarly or differently with these outcomes could inform more targeted prevention approaches.

Third, although some studies have examined social determinants of suicide and NSSI in youth (Hielscher et al., 2024; McDermott et al., 2018; Wang and Wu, 2021), much of the social determinants literature has focused on adults (Gallagher et al., 2025; Na et al., 2025). This creates challenges in translating findings to youth populations because social determinants are often operationalised in ways that are less relevant for young people (e.g. household income is arguably more relevant than personal income for youth living with parents or guardians), which limits our understanding of the most important social determinants for youth.

### The present study

Our study aimed to determine which of the social determinants listed in the NSPS model had the strongest associations with 12-month suicidal ideation and NSSI among Australian youth aged 16–24 years. Using nationally representative data from the cross-sectional 2020–2022 National Study of Mental Health and Wellbeing (NSMHW), we conducted population-weighted logistic regressions to examine the relative contribution of each social determinant (over and above the others) to the odds of engaging in (1) suicidal ideation and (2) NSSI. We did not make specific hypotheses about which determinants would have the largest association with our outcomes of interest.

## Method

### Data and sample

We used the most recently released data set from the NSMHW, a cross-sectional study funded by the Australian Government Department of Health and Aged Care and administered by the Australian Bureau of Statistics (ABS). The NSMHW aimed to examine the prevalence of mental health conditions and suicidal thoughts and behaviours among Australians (see Arya et al., 2024 for results on prevalence), as well as patterns of mental health service use and effectiveness. Detailed information about the methodology is published elsewhere (ABS, 2023a). Briefly, a stratified area sampling design was used to select private dwellings across urban and rural areas in all Australian states and territories. For each selected dwelling, one person was randomly selected to participate in a face-to-face interview. It is worth noting that this design entirely excluded people who were residing in (1) ‘special’ (i.e. non-private) dwellings, (2) very remote areas and (3) discrete Aboriginal and Torres Strait Islander communities. Due to COVID-related disruptions, data were collected in two cohorts: December 2020–July 2021 (*N* = 5554) and December 2021–October 2022 (*N* = 10,339), resulting in a combined sample of 15,893 respondents aged 16–85 years (response rate: 52.0%).

Our participants constituted a subsample of this full data set: the 1620 people aged 16–24 years at the time of data collection. Participants had a mean age of 20.32 years (SD = 2.56) and included 791 men/boys (48.83%), 806 women/girls (49.75%) and 23 people who identified as non-binary or chose not to disclose their gender identity (1.42%). We obtained ethical approval for our analysis from the Human Research Ethics Committee at the Australian National University (Protocol #2024/0889).

### Survey instrument

The NSMHW included questions about mental health conditions, health service use, suicidal thoughts and behaviours, and demographic and socioeconomic characteristics, drawing from the questions in the World Mental Health Survey Initiative version of the Composite International Diagnostic Interview (version 3.0). In this study, we used measures of suicidal ideation and NSSI in the past 12 months (i.e. ‘Did you seriously think about taking your life any time in the past 12 months?’; ‘Have you intentionally harmed yourself in the last 12 months?’). Questions about 12-month suicidal thoughts and behaviours were only administered to participants who endorsed lifetime incidence of the corresponding thought or behaviour. We focused on 12-month suicidal ideation and NSSI, with the rationale that it would more accurately capture associations between these outcomes and our selected social determinants variables, which primarily related to current or recent circumstances.

We used questions from the demographic and socioeconomic sections of the survey to capture our key social determinants predictors. These included (1) current weekly household income, (2) current receipt of government pensions or allowances, (3) current engagement in at least part-time work or study, (4) lifetime history of homelessness, (5) number of recent household financial stress issues, (6) current residence in an urban (vs regional or remote) area and (7) current level of social connectedness (see Table 1 for operationalisations, rationale and evidence). The NSPS also contains the following social determinants that we were unable to find relevant and youth-appropriate measures in the NSMHW data set: working life conditions, structural equality, early childhood development and access to quality health care.

**Table 1.** Operationalisations of NSPS social determinants using NSMHW variables.

NSPS social determinant	Indicator	Rationale for predictor selection	NSMHW operationalisation/coding	Evidence for relationship with suicide and self-harm
Income and social protection	1. Household income	Household income is likely more relevant than personal income for youth aged 15–24, given that most young people in this population live with family (AIHW, 2021). Family poverty has also been used previously as a predictor of youth suicide (Wang and Wu, 2021).	Variable: Total current weekly household income from all sources. Each unit = an additional AU$1000 of household income per week.	Low income is associated with increased risk of suicide and self-harm (Lodebo et al., 2017; Qin et al., 2022).
	2. Income from government pension or allowance payments	Focused on young people’s receipt of pension/allowance payments, rather than household receipt, as this was theorised to more directly affect the life of the young person (compared to whether, for example, their parents receive such payments).	Variable: All sources of personal income. 0 = not receiving any government pension or allowance payments as part of current income 1 = receiving one or more government pension or allowance payments.	Government pension or allowance payments for unemployed people are protective against suicide; less research on self-harm (Shand et al., 2022).
Employment and job security; education	3. Engaged in at least part-time work or study	Reflects category often used to describe at-risk youth: people not in education, employment or training (NEET). Part-time engagement in either work or education is more meaningful for youth, who are often navigating school, part-time work and/or tertiary education. Highest level of educational attainment is a more commonly used indicator but is less relevant for youth, as many are still studying.	Variable: Engagement in education or employment. 0 = not currently engaged in part-time work or study 1 = currently engaged in at least part-time work or study.	NEET youth are at higher risk of suicide and self-harm (Gariépy et al., 2022; Haugland and Stea, 2022).
Housing and basic amenities	4. Ever experienced homelessness	Unable to assess current homelessness due to NSMHW design (excluding people not residing in a private dwelling), so chose available variable measuring whether participants had ever experienced homelessness. Homelessness during childhood may be particularly detrimental (Murran and Brady, 2023).	Variable: Whether ever been without a ‘permanent place to live’. 0 = never been without a permanent place to live 1 = have been without a permanent place to live.	People experiencing homelessness have increased risk of suicide and self-harm (Ayano et al., 2019; Clements et al., 2022).
	5. Number of household financial stress issues	Captures acute financial stress, beyond income measures. Many of the 11 financial stress issues asked about in the NSMHW also relate to a lack of access to basic amenities (e.g. going without meals, unable to heat or cool home).	Variable: Type of household cash flow problems in previous 12 months. Each unit (1–11) = household experience of one additional ‘cash flow problem’ in the last 12 months.	Greater financial stress predicts increased risk of suicide and self-harm thoughts and behaviours (Paul and Fancourt, 2022; Roelfs and Shor, 2023).
Environment	6. Residence in an urban or regional/remote location	Environment operationalised as urban vs. regional/remote based on examples given in similar social determinants models (Pirkis et al., 2024).	Variable: Accessibility/Remoteness Index of Australia, which classifies remoteness according to a person’s relative access to services (ABS, 2023b). 0 = residing in a regional or remote area 1 = residing in an urban area (major cities).	Some research has found residence in rural (vs urban) areas increases risk of (completing) suicide, especially for men (Barry et al., 2020). Other reviews show increased risk for urban residents (Satherley et al., 2022). Research on self-harm also shows mixed findings (Harriss and Hawton, 2011; Wang et al., 2022).
Social inclusion and non-discrimination	7. Social connectedness	Chosen based on research indicating that the extent to which a person sees themselves as a member of a group or community is far more impactful, than, for example, the number of social ties that a person has (Mehrpour et al., 2024).	Variable: Single item^a^ measuring current sense of being part of a group or community (1 = *Poor* to 5 = *Excellent*). Mean centred (M = 2.71) before entry into the regression models.	Social connectedness protects against suicide and NSSI; social isolation and loneliness increase risk (Blacutt and Ammerman, 2025; Blázquez-Fernández et al., 2023; McClelland et al., 2020; Wu et al., 2013).

a Six other social connectedness items from the Living in the Community Questionnaire (Australian Mental Health Outcomes and Classification Network, 2015) were available, but we chose not to combine them into one scale due to significant variation in the content and response scale of the items.

### Analytic strategy

Our key analyses were two population-weighted multivariable logistic regressions conducted in the secure ABS Datalab environment using Stata (Version 17.0) predicting 12-month suicidal ideation and NSSI, with all social determinant predictors added simultaneously. Following conventional standards, we considered results of these regressions significant if the *p* value was below <0.05. Population weighting was applied using the variable developed by the ABS to ensure that the results reflected the broader Australian population aged 16–24 years, rather than the sample (see ABS, 2023a for full details of ABS population weighting methods). The suicidal ideation and NSSI outcome variables were created by assigning a value of 1 to people who reported suicidal ideation/NSSI in the past 12 months and a zero to everyone else (unless coded as missing). Coding for the social determinants variables is detailed in Table 1. For all variables, participants who did not wish to answer the relevant questions or said that they did not know were coded as missing. All other data were retained, except for one extreme outlier removed from the household income variable before analysis. Given the large sample, relatively low proportion of missing data and the use of population weighting, missing data were handled using listwise deletion (*N* = 1329 for suicidal ideation analyses; *N* = 1308 for NSSI analyses).

## Results

Population-weighted proportions and frequencies calculated from sample-level descriptives were as follows: an estimated 7.57% of young Australians (approximately 117,626 people) experienced suicidal ideation in the 12 months before the study, and 7.12% (approximately 164,704 people) engaged in NSSI. Population-weighted correlations are reported in Table 2. These indicated that suicidal ideation and NSSI were moderately positively correlated with each other. Both outcomes were negatively correlated with work/study engagement and social connectedness, positively correlated with homelessness and financial stress, and non-significantly correlated with urban residence. NSSI was also negatively correlated with household income and positively correlated with receiving government pensions/allowances, but suicidal ideation was not.

**Table 2.** Correlations between key study variables (population weighted).

Variable	1	2	3	4	5	6	7	8
1. 12-month suicidal ideation	1.00							
2. 12-month NSSI	0.52***	1.00						
3. Household income	−0.04	−0.05*	1.00					
4. Income from government pensions or allowances	0.06	0.08*	−0.15***	1.00				
5. Engaged in at least part-time work or study	−0.11*	−0.12*	0.13***	−0.27***	1.00			
6. Ever experienced homelessness	0.13**	0.16**	−0.09***	0.15***	−0.09*	1.00		
7. Number of household financial stress issues	0.09*	0.20***	−0.15***	0.19***	−0.16**	0.27***	1.00	
8. Residence in an urban location	−0.004	0.01	−0.04	0.001	0.05	−0.04	−0.07*	1.00
9. Social connectedness	−0.25***	−0.28***	0.10***	−0.12***	0.16***	−0.19***	−0.16***	0.06

* *p* < 0.05; **p < 0.01; ***p < 0.001.

### Regression results

Multivariable regression results are presented in Tables 3 and 4. Social connectedness was the only statistically significant predictor across both the NSSI and suicidal ideation models. The odds ratios for social connectedness indicated that a one-unit increase above the mean in the extent to which participants felt part of a group or community reduced the odds of suicidal ideation by 52% and NSSI by 55% (odds ratios = 0.48 and 0.45, respectively). Apart from social connectedness, only two other predictors were statistically significant. Specifically, having ever experienced homelessness more than doubled the odds of suicidal ideation (odds ratio = 2.16), but was not a statistically significant predictor of NSSI. Financial stress was a statistically significant predictor of NSSI but not suicidal ideation, with each additional stressor increasing the odds of NSSI by 34% (odds ratio = 1.34). Notably, no other social determinant (i.e. household income, receipt of government pension or allowance payments, work/study engagement and urban residence) was statistically significant in either model. Examination of the size of the odds ratios indicates that some non-significant effects may still be important, especially experience of homelessness on NSSI, and work/study engagement and urban residence on both outcomes. Variance inflation factors (VIFs) and low correlations between predictors (see Table 2) indicated no evidence of multicollinearity (mean VIF of each model = 2.30 and 2.31, largest VIF ~5; Kalnins and Praitis Hill, 2025).

**Table 3.** Population-weighted logistic regression model predicting 12-month suicidal ideation in 16- to 24-year-olds.

Predictor	Odds ratio	SE	95% confidence interval	*p*
1. Household income	0.99	0.07	[0.87, 1.13]	0.878
2. Income from government pension or allowance payments	0.84	0.50	[0.26, 2.68]	0.773
3. Engaged in at least part-time work or study	0.52	0.26	[0.20, 1.38]	0.190
**4. Ever experienced homelessness**	**2.16**	**0.76**	**[1.08, 4.33]**	**0.029**
5. Number of financial stress issues	1.09	0.09	[0.93, 1.28]	0.288
6. Residence in an urban location	1.20	0.38	[0.64, 2.24]	0.574
**7. Social connectedness**	**0.48**	**0.06**	**[0.39, 0.61]**	**<** **0.001**

Model Pseudo *R*^2^ = 0.12. SE = Robust standard error. Model *N* = 1329. Wald *X*^2^(7) = 68.17, *p* < 0.001. Statistically significant results are given in bold (i.e. where *p* < 0.05).

**Table 4.** Population-weighted logistic regression model predicting 12-month NSSI in 16- to 24-year-olds.

Predictor	Odds ratio	SE	95% confidence interval	*p*
1. Household income	0.99	0.06	[0.89, 1.12]	0.993
2. Income from government pension or allowance payments	0.92	0.36	[0.43, 1.96]	0.821
3. Engaged in at least part-time work or study	0.69	0.32	[0.27, 1.74]	0.427
4. Ever experienced homelessness	1.93	0.83	[0.83, 4.48]	0.128
**5. Number of financial stress issues**	**1.34**	**0.14**	**[1.09, 1.64]**	**0.005**
6. Residence in an urban location	1.41	0.52	[0.69, 2.90]	0.344
**7. Social connectedness**	**0.45**	**0.05**	**[0.36, 0.57]**	**<** **0.001**

Model Pseudo *R*^2^ = 0.17. SE = Robust standard error. Model *N* = 1308. Wald *X*^2^(7) = 81.24, *p* < 0.001. Statistically significant results are given in bold (i.e. where *p* < 0.05).

## Discussion

Using a large, nationally representative sample of Australian youth, we cross-sectionally examined which of the social determinants listed in *the National Suicide Prevention Strategy 2025–2035* (NSPS) social determinants model (that were operationalisable using available data) was most strongly related to 12-month suicidal ideation and NSSI. Results revealed that social connectedness was the only social determinant that reduced the odds of both suicidal ideation and NSSI (odds ratios = 0.48 and 0.45, respectively). In addition, having ever experienced homelessness more than doubled the risk (odds ratio = 2.16) of suicidal ideation, but was not associated with NSSI, and experiencing financial stress increased the risk of NSSI by a third (odds ratio = 1.34), but was not associated with suicidal ideation. The other social determinants were statistically non-significant.

Our findings align with extensive theorising and empirical evidence emphasising the importance of social relationships for health and in preventing suicide and NSSI (Blázquez-Fernández et al., 2023; De Silva et al., 2005; Durkheim, 1997; Haslam et al., 2018; Jetten et al., 2017; McClelland et al., 2020; Motillon-Toudic et al., 2022; Shoib et al., 2023; Van Orden et al., 2010). In particular, in demonstrating the importance of social connectedness – which could be conceptualised as the opposite of thwarted belongingness – our findings support the Interpersonal Theory of Suicide discussed above (Van Orden et al., 2010). Our findings also speak to the value of the social identity approach to health (Haslam et al., 2018; Jetten et al., 2017), which positions social *group* connections as especially crucial for mental and physical health. Given that minimal existing research has applied the social identity approach to suicide and NSSI, this may be a promising avenue for research. Such research might examine the extent to which people see themselves as part of meaningful social groups (rather than simply the number of friends or amount of general social support they have), which would also extend prior work demonstrating that it is the *quality* rather than quantity of social interactions that matters most for protecting against NSSI (Blacutt and Ammerman, 2025).

We found different association patterns for suicidal ideation and NSSI, such that social connectedness was negatively associated with both outcomes, while experience of homelessness and financial stress were positively associated with only suicidal ideation and NSSI, respectively. This finding aligns with evidence outlined earlier that suicide and NSSI have distinct risk pathways (Mars et al., 2014) and may reflect the theorised functions of engaging in suicidal thoughts and behaviours versus NSSI. Suicidal thoughts and behaviours are theorised to be, at least in part, a response to chronic feelings of hopelessness and social isolation (Beck et al., 1990; Van Orden et al., 2010), both of which are highly prevalent in people experiencing homelessness (Hausam et al., 2025; Lachaud et al., 2024). Conversely, NSSI has been found to serve as a strategy for managing acute distress, including financial stress (Barnes et al., 2016; Edmondson et al., 2016; Nock and Prinstein, 2004).

Finally, it is worth considering possible reasons for the non-significance of the other social determinants examined. One possibility is that these social determinants are less relevant for youth, indicating the need for a youth-specific model. Another possibility is that the relationship between some of the social determinants we examined and suicidal ideation/NSSI is not straightforwardly linear, requiring more complex modelling (see Congdon, 2012; Hielscher et al., 2024; Hsu et al., 2015). A third, more methodological explanation is that our findings reflect challenges in operationalising the social determinants, owing to both the variables available in the NSMHW and the lack of clear operational definitions in the NSPS model (e.g. the ‘environment’ social determinant in the NSPS). Ultimately, this lack of definitional clarity (also noted by Gallagher et al., 2025) needs to be addressed to support improved quality and consistency of empirical work in this area.

Worth also noting is that several social determinants that were non-significant in multivariable models nevertheless had substantial effect sizes (e.g. work/study engagement reduced odds by 48% for suicidal ideation) and statistically significant bivariate associations with suicidal ideation and NSSI. This may be partially explained by the fact that social connectedness was correlated with nearly all other social determinants (as was financial stress), which may have affected the strength of their associations in the multivariable model. This highlights the complex, interrelated nature of social determinants and the possibility that interventions addressing social connectedness may have positive effects on other social determinants (and vice versa).

### Implications for policy

Our findings have important policy implications, particularly for implementing and refining the NSPS. First, we find distinct social determinants associated with suicidal ideation (social connectedness and experience of homelessness) and NSSI (social connectedness and financial stress), indicating the need to consider the risk and protective factors for suicidal ideation and NSSI separately (rather than putting them in the same ‘box’, as in the NSPS model).

Second, our findings highlight the potential effectiveness of key NSPS prevention strategies and recommended actions, in particular, strategies 3 (increasing economic security) and 4 (enhancing social inclusion). For example, under strategy 3, our findings support the benefit of strengthening ‘*connections between financial counselling, mental health and suicide prevention supports to meet the holistic and complex needs of people experiencing financial distress*’ (Action 3.2.d: 31) for reducing NSSI. Our findings also support the benefit of ‘*equitable and inclusive access to safe, secure and affordable housing across the spectrum of housing and housing services, including homelessness services* ...’ (Action 3.2.e: 31) for reducing suicidal ideation. Under strategy 4, our findings support the suggestion to ‘*implement and evaluate programs to build social connectedness and a sense of belonging, and improve relationships, including through funding community-based programs*’ (Action 4.2a: 35). Our findings indicate that this action may help to reduce both suicidal ideation and NSSI in young people, although longitudinal cohort studies are required to confirm this.

Social connectedness interventions could, therefore, be considered one of the key next steps in youth suicide and NSSI prevention efforts. Several evidence-based approaches show promise, including (1) social prescribing programmes, which connect people to community-based activities and services (Dash et al., 2025); (2) community-based initiatives, such as Neighbour Day, an annual event that encourages people to connect with their neighbours (Relationships Australia, 2025; also highlighted as an activity to build on in the NSPS); and (3) psychosocial interventions that address social and psychological risk factors through structured content and activities, which have already demonstrated efficacy for preventing youth suicide and NSSI (Calear et al., 2016; Nawaz et al., 2024; Robinson et al., 2018). When designing and implementing social connectedness interventions, it is important to consider that young people experiencing suicidal distress are not a homogeneous group, and some may experience additional risk factors or practical constraints that limit their meaningful participation (e.g. Neighbour Day may be less relevant for a young person experiencing homelessness). That is, these interventions will not be well-implemented if those they intend to reach cannot meaningfully participate in them.

Our findings also have implications for suicide and NSSI prevention efforts internationally. The potentially protective role of social connectedness aligns with international evidence (Calati et al., 2019) and recent calls for urgent action to combat loneliness (World Health Organization, 2025). Similarly, the differential associations of social determinants with suicidal ideation and NSSI are consistent with international evidence of distinct pathways to different self-harm outcomes (Mars et al., 2014) and suggest that differentiated prevention approaches may be beneficial outside Australia. More broadly, our study demonstrates the value of empirically testing frameworks proposed in national prevention strategies to identify priority intervention targets. Given that the World Health Organization (2018) explicitly encourages governments to learn from other countries’ national suicide prevention strategies, we hope that our insights into the NSPS will assist other countries in developing or refining their own national strategies.

### Strengths, limitations and directions for future research

A major strength of our study is the use of a large, nationally representative, and recent data set, allowing us to generate strong evidence about the relative importance of social determinants on suicidal ideation and NSSI in Australian youth. A key limitation is the use of cross-sectional data. While such data provide important insights into key social determinants associated with suicidal ideation and NSSI, as guided by the NSPS framework, future research is needed to establish temporal precedence and the direction of these effects. Specifically, further research is needed to determine whether social connectedness reduces suicidal ideation/NSSI or if the absence of suicidal ideation/NSSI allows for greater social connectedness. Such research could use prospective longitudinal designs to track youth over time and examine whether naturally occurring changes in social connectedness (or other social determinants) precede changes in suicidal ideation/NSSI. There are numerous ethical and practical barriers to manipulating social determinants and suicidal distress; however, randomising youth to receive interventions targeting key social determinants and examining the effect on suicidal ideation/NSSI over time could also provide stronger evidence of the directionality of effects.

Other limitations include the use of single-item measures (particularly for social connectedness), and the exclusion criteria of the NSMHW, which impacted our ability to generalise to people *currently* experiencing homelessness, located in very remote areas, and living in discrete Aboriginal and Torres Strait Islander communities, all of which are high-risk populations (AIHW, 2025c; Ayano et al., 2019). The social determinant variables available also had varying reference periods (e.g. social connectedness referred to the past 4 weeks, financial stress to 12 months), which complicates direct comparisons of effect sizes.

Our focus on assessing the relative importance of social determinants meant that we did not assess individual risk factors (e.g. mental health, trauma) in our models. We were also unable to model all social determinants in the NSPS due to a lack of available variables in the NSMHW and some unclear definitions in the NSPS. Future research could test combined social and individual risk factor models to disentangle the effects of social determinants over and above their association with individual risk factors (e.g. links between experience of homelessness and the individual risk factors of trauma, mental health conditions and substance use; Arnautovska et al., 2014; Ayano et al., 2019).

Future work should also seek to develop a well-defined, youth-specific social determinants model. This could follow the example of McDermott et al. (2018), who used qualitative methods to identify relevant social determinants of suicide among LGBTQIA+ youth before quantitatively testing them (see also child-centred qualitative methods used by Bessell et al., 2024). Quantitative tests of such models could also use person-centred statistical analyses (e.g. latent class analysis; Afroz et al., 2023) to investigate how social determinants cluster among people.

Finally, the NSMHW data were collected during the COVID-19 pandemic, which may have impacted the generalisability of the results. In particular, the pandemic created unique circumstances for many of the key social determinants we studied. For example, infection control measures such as lockdowns increased loneliness and detrimentally affected young people’s mental health (Killgore et al., 2020; Magson et al., 2021). In addition, a much larger portion of the population received government allowances (e.g. 14% of Australians aged 16–64 years in March 2020; 20% in June 2020; AIHW, 2025b), and usual payment rates were supplemented for part of 2020 (Australian Government: Services Australia, 2020). Future research should, therefore, investigate whether these findings replicate post-pandemic.

## Conclusion

Our study provided the first empirical test of the NSPS social determinants model in Australian youth. Social connectedness was the only determinant associated with both suicidal ideation and NSSI, reducing the odds by more than half. Experience of homelessness and financial stress were also identified as risk factors for suicidal ideation and NSSI, respectively, with experience of homelessness having a particularly detrimental effect. These findings support NSPS prevention actions aimed at strengthening social inclusion and economic security. Unlike many suicide risk factors, which are difficult or impossible to change, social connectedness can be cost-effectively modified through existing, evidence-based interventions. We, therefore, recommend that testing and implementing social connectedness interventions be considered one of the next steps in youth suicide and NSSI prevention efforts.
